# Ultrasensitive discrimination of volatile organic compounds using a microfluidic silicon SERS artificial intelligence chip

**DOI:** 10.1016/j.isci.2023.107821

**Published:** 2023-09-02

**Authors:** Haiting Cao, Huayi Shi, Jie Tang, Yanan Xu, Yufan Ling, Xing Lu, Yang Yang, Xiaojie Zhang, Houyu Wang

**Affiliations:** 1 Suzhou Key Laboratory of Nanotechnology and Biomedicine, Institute of Functional Nano & Soft Materials (FUNSOM), Soochow University, Suzhou, Jiangsu 215123, China; 2 State Key Laboratory of Radiation Medicine and Protection, School of Radiation Medicine and Protection, Collaborative Innovation Center of Radiological Medicine of Jiangsu Higher Education Institutions, Soochow University, 199 Renai Road, Suzhou 215123, China; 3 Department of Thoracic Surgery, Shanghai Pulmonary Hospital, School of Medicine, Tongji University, Shanghai 200433, China; 4 Department of Experimental Center, Medical College of Soochow University, Suzhou, Jiangsu 215123, China

**Keywords:** Fluidics, Nanoscience, Nanotechnology

## Abstract

Current gaseous sensors hardly discriminate trace volatile organic compounds at the ppt level. Herein, we present an integrated platform for simultaneously enabling rapid preconcentration, reliable surface-enhanced Raman scattering, (SERS) detection and automatic identification of trace aldehydes at the ppt level. For rapid preconcentration, we demonstrate that the nozzle-like microfluidic concentrator allows the enrichment of rare gaseous analytes by five-fold in only 0.01 ms. The enriched gas is subsequently captured and detected by an integrated silicon-based SERS chip, which is made of zeolitic imidazolate framework-8 coated silver nanoparticles grown *in situ* on a silicon wafer. After SERS measurement, a fully connected deep neural network is built to extract faint features in the spectral dataset and discriminate volatile organic compound classes. We demonstrate that six kinds of gaseous aldehydes at 100 ppt could be detected and classified with an identification accuracy of ∼80.9% by using this platform.

## Highlights

• A platform allows preconcentration, detection, and identification of trace VOCs
• The platform is composed of a concentrator and an SERS chip
• Six kinds of gaseous aldehydes of 100 ppt could be discriminated

## Graphical abstract

**Figure:**
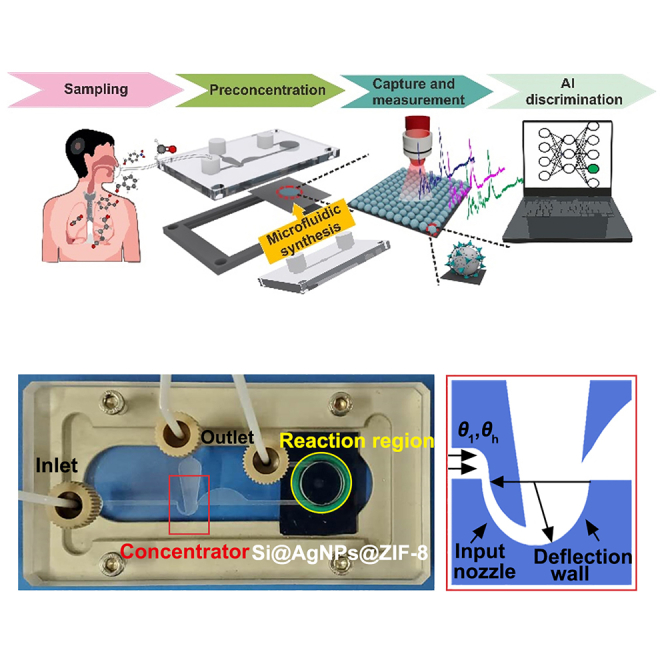

## Introduction

Volatile organic compounds (VOCs), known as potentially informative endogenous signaling molecules, are widely found in human exhaled breath, blood, urine and sweat. Impressively, VOCs have been proven to be tightly associated with various pathophysiological processes.^1^^,^^2^^,^^3^^,^^4^^,^^5^^,^^6^ For instance, the levels of several VOCs in the breath of lung cancer patients range from ∼10 to 200 ppb (parts per billion), approximately one or two orders of magnitude higher than those of healthy individuals.^7^^,^^8^ Therefore, breath testing of VOCs for disease diagnostics has received increasing attention due to its rapid and noninvasive merits.^9^^,^^10^^,^^11^ However, conventional approaches of breath analysis using gas chromatography/mass spectrometry (GC‒MS), ion flow tube mass spectrometry, laser absorption spectrometry, and infrared spectroscopy are generally time-consuming and laborious and require professional staff to operate expensive and complex instruments.^2^^,^^12^^,^^13^ As such, it places a giant burden on clinical use, especially for developing countries, where healthcare systems are underfunded and modern diagnostic instruments are limited. For point-of-care (POC) testing, there has been a growing trend to develop gaseous sensors, such as colorimetric sensors, e-nose/e-tongue sensors, reflectance sensors, field effect transistor (FET) sensors, and surface-enhanced Raman scattering (SERS) sensors.^14^^,^^15^^,^^16^^,^^17^^,^^18^^,^^19^^,^^20^^,^^21^^,^^22^^,^^23^

It is worthwhile to note that the state-of-the-art gaseous sensors hardly discriminate trace VOCs at the ppt (part per trillion) level. Most gas sensors merely monitor ppm (part per million) or ppb-level VOCs due to their mediocre sensitivity.^14^^,^^15^^,^^16^^,^^17^^,^^18^ For SERS sensors, despite the prominent surface plasmon resonance (SPR) effect (e.g., enhancement factor (EF) of up to ∼10^12^^,^^13^^,^^14^),^24^^,^^25^^,^^26^^,^^27^ their detection sensitivity is attenuated by three bottlenecks. First, rapid online preconcentration techniques compatible with SERS substrates to effectively improve the detection sensitivity are still few.^3^^,^^4^ Second, distinguished from liquid-interfacial analysis,^28^^,^^29^ in gas-interfacial analysis, gaseous molecules diffuse more rapidly than liquid and solid molecules because there is more free space between molecules. Because of the fast diffusion rate, there is insufficient contact between the analyte gas and the solid matrix. As a result, most of the gaseous analyte is washed away by the prevailing gas stream in most situations, leaving only a small percentage of molecules adsorbed to the SERS substrate. Although some porous structures (e.g., metal organic frameworks (MOFs)) coated onto SERS substrates can slow the diffusion rate of VOCs,^25^^,^^30^^,^^31^^,^^32^^,^^33^^,^^34^ the relatively thick coated shell (e.g., ∼50–150 nm) is still detrimental to detection sensitivity. The majority of VOC molecules might be non-specifically adsorbed onto the scaffold sites of the MOF shell, which would block the holes of MOFs.^16^^,^^35^ Third, VOCs generally display subtle differences in their corresponding Raman spectra due to their similar molecular structures (e.g., alcohols, aldehydes, and aromatic compounds), and in the case of trace VOCs, the differences could be easily masked by background noise.^30^^,^^31^^,^^32^^,^^33^^,^^34^^,^^35^

**Figure 1:**
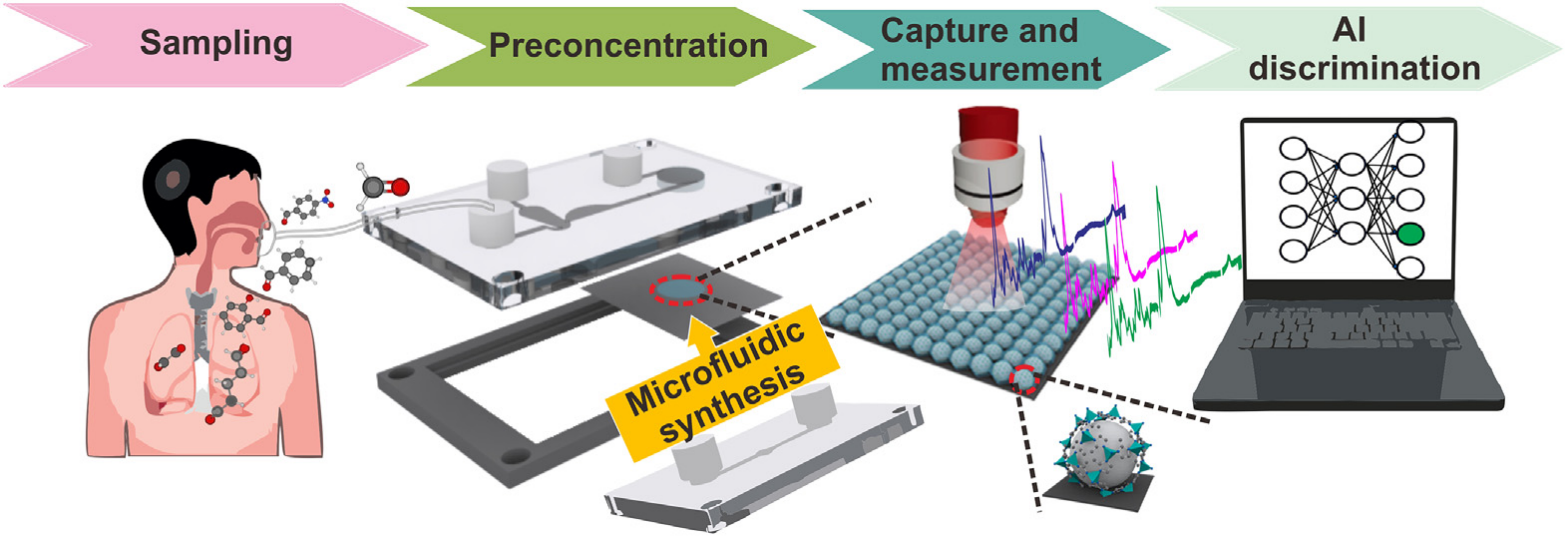
Schematic illustration of an SERS-active microfluidic device combined with artificial intelligence (AI) for the discrimination of VOCs The platform is composed of a nozzle-like microfluidic concentrator, ZIF-8-coated SERS chip and DNN algorithm. A ZIF-8-coated SERS chip is synthesized by microfluidic galvanic deposition coupled with microfluidic MOF self-assembly.

Herein, we present a novel integrated sensing platform for rapid preconcentration, reliable detection and automatic identification of trace aldehyde VOCs down to the ppt level. The developed platform consists of three key components: a nozzle-like microfluidic concentrator for VOC preconcentration, a silicon-based SERS chip made of 10 nm thick MOF shell-coated plasmonic NPs on a silicon wafer for the capture and detection of VOCs, and deep neural networks (DNNs) for SERS data processing (Figure 1). Compared with other reported VOC sensors, this developed platform features several advantages: (1) the nozzle-like microfluidic concentrator enables quick online preconcentration of VOCs (5-fold enrichment in only 0.01 ms) based on their molecular weights; (2) the silicon-based chip is prepared via microfluidic galvanic deposition coupled with microfluidic MOF self-assembly, yielding a homogeneous SERS substrate coated with a thin MOF shell and thus guaranteeing reliable SERS measurement; and (3) online preconcentration and a thin MOF shell-coated silicon SERS chip coupled with a fully connected DNN allows ultrasensitive discrimination of six kinds of gaseous aldehydes with a limit of detection down to 100 ppt and with an identification accuracy of ∼80.9%.

## Results

### Design and fabrication of microfluidic SERS device

**Figure 2:**
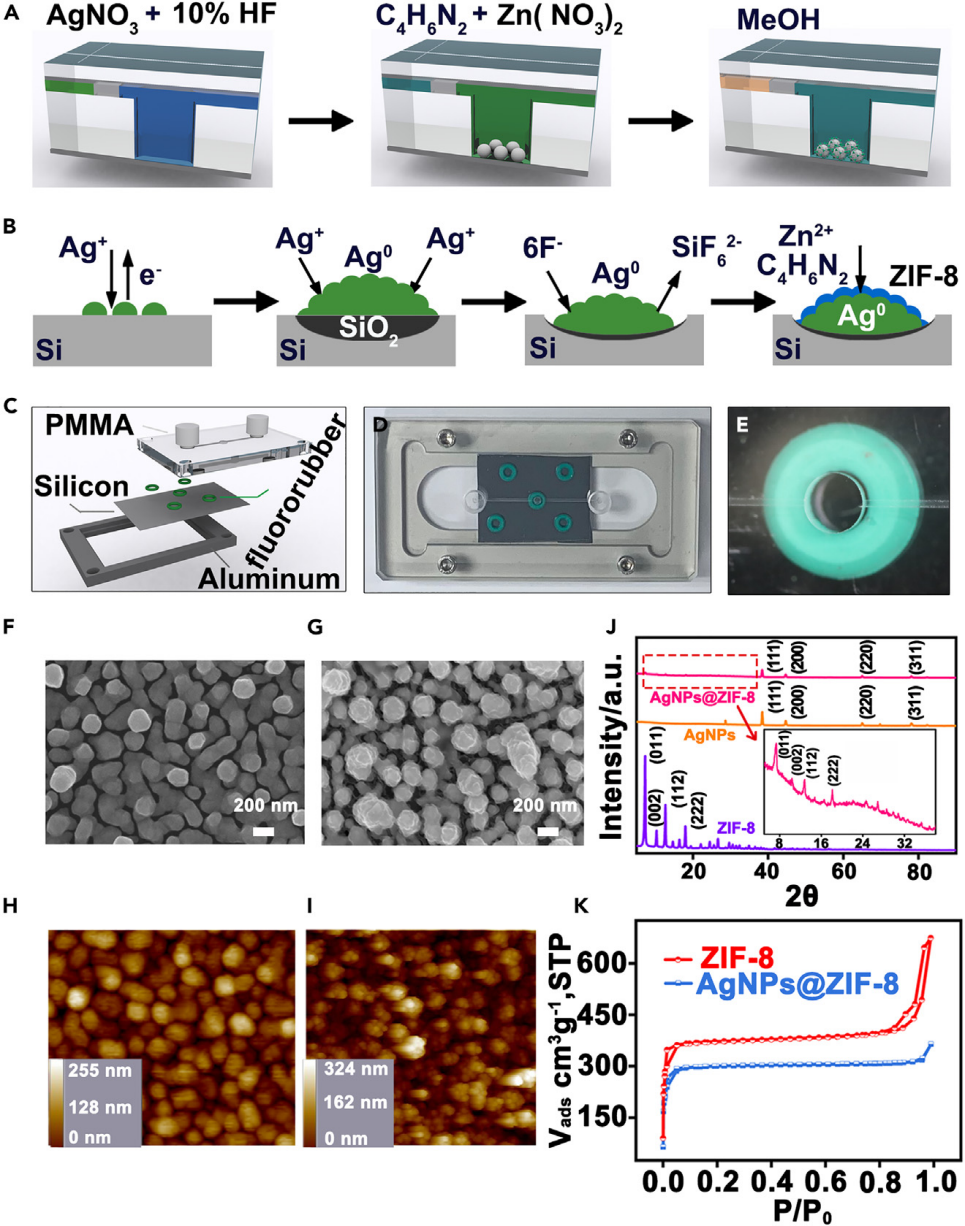
Synthesis and characterization of Si@AgNPs@ZIF-8 (A) Schematic illustration of the synthesis of Si@AgNPs@ZIF-8 in the microfluidic system. (B) Schematic illustration of the mechanisms of microfluidic galvanic deposition (GD) and microfluidic ZIF-8 self-assembly. (C) Schematic design of the microfluidic reactor. (D) Real photo of the microfluidic chip. (E–I) (E) Zoomed-up top view of the reaction chamber. SEM images of AgNPs@Si (F) and Si@AgNPs@ZIF-8 (G). AFM images of AgNPs@Si (H) and Si@AgNPs@ZIF-8 (I). (J) XRD patterns of AgNPs@ZIF-8 (pink line), AgNPs (yellow line) and ZIF-8 (purple line). The inset is a partially enlarged XRD diffractogram of AgNPs@ZIF-8. (K) N_2_ adsorption-desorption isotherms of ZIF-8 (red line) and AgNPs@ZIF-8 (blue line).

We developed a microfluidic synthetic strategy for the preparation of MOF-shell-coated SERS chips (25 mm × 25 mm), which were made of zeolitic imidazolate framework-8 (ZIF-8)-coated silver nanoparticles (AgNPs) grown *in situ* on silicon wafers (Si@AgNPs@ZIF-8). Experimentally, hydrogen fluoride (HF) solution (10%, v/v) was first injected into the microchannel at a flow rate of 6 μL/min for 30 min to form Si-H bonds at the silicon interface in the microchamber. Then, nitrogen was injected into the channel to form an air column of ∼1–3 cm length to separate the fore HF solution and the latter reaction solution. Afterward, silver nitrate (AgNO_3_) solution containing HF (10%, v/v) was pumped into the channel at a flow rate of 6 μL/min for several minutes (Figure 2A). When the reaction solution contacted HF-treated silicon, the galvanic deposition (GD) reaction was triggered. As a result, homogeneous AgNPs were grown *in situ* on a silicon wafer (AgNPs@Si) by microfluidic GD. Next, ZIF-8 precursors of zinc nitrate hexahydrate (Zn(NO_3_)_2_·6H_2_O) and 2-methylimidazole (C_4_H_6_N_2_) dissolved in methanol were pumped into the microchannel at a flow rate of 6 μL/min to form the ZIF-8 shell.

The mechanism of synthesis of Si@AgNPs@ZIF-8 is illustrated in Figure 2B. Typically, Ag nuclei were formed in the vicinity of the reactive silicon surface when Ag^+^ received electrons from the valence band (VB) of silicon. Meanwhile, SiO_2_ was formed underneath the AgNPs and etched away by HF solution. As a result, pits were formed immediately beneath the Ag deposits, and the AgNPs gradually sank into the as-resultant pits when they were formed.^36^^,^^37^^,^^38^^,^^39^ Next, ZIF-8 shells were grown *in situ* on the AgNP surface, in which Zn^2+^ was the metal center and 2-methylimidazole was the organic ligand. The homemade microfluidic device was integrated with an upper polymethyl methacrylate (PMMA) chip, middle silicon wafer and bottom aluminum alloy bracket (Figure 2C). Figures 2D and 2E display the real photo of the homemade microfluidic device and the magnified top view of the microchamber, respectively.

Different from uncontrollable synthetic reactions in the bulk/macro system,^36^^,^^37^^,^^38^^,^^39^ Ag nucleation, the growth of AgNPs and the formation of the ZIF-8 shell could be effectively controlled in the microfluidic system since the transportation equilibrium of reacting species can be rapidly achieved in the designed microfluidic system.^40^ As simulated by COMSOL Multiphysics software (ver. 5.3; COMSOL, Inc., Burlington, MA, USA) (Figure S1), the homogeneous distribution of Ag^+^ or ZIF-8 precursors (e.g., Zn^2+^) in the microfluidic system can be quickly reached within only 5 s, while the concentration gradient of Ag^+^ or ZIF-8 precursors was still obvious in the bulk system even when the simulation time was up to 50 s. As a consequence, SERS substrates with more homogeneous size, spatial distribution and thinner shell were achieved by microfluidic synthesis (Figure S2).

We systematically characterized the as-prepared Si@AgNPs@ZIF-8 by scanning electron microscopy (SEM), atomic force microscopy (AFM), X-ray diffraction (XRD), and BET adsorption-desorption isotherms. As shown in the SEM images (Figure 2F), AgNPs with smooth surface topography were uniformly grown on the silicon surface. After coating with ZIF-8, the surface of the nanostructure became rough (Figure 2G). AFM images further confirmed the core-shell structure of the as-prepared SERS chip (Figures 2H and 2I). The size distribution of AgNPs and AgNPs@ZIF-8 was 168.35 ± 18.47 nm and 178.35 ± 20.24 nm, respectively, by statistical analysis of 100 nanoparticles (Figure S3). As such, the average thickness of the ZIF-8 shell was approximately 10 nm. Of note, such thickness was only one-fifth to one-tenth of MOF shells in other counterparts.^16^^,^^41^ As shown in the XRD patterns (Figure 2J), four characteristic diffraction peaks centered at 7.3°, 10.3°, 12.7° and 18.0° were assigned to the (011), (002), (112), and (222) planes of ZIF-8 in pure ZIF-8 and AgNPs@ ZIF-8, respectively. For AgNPs@ZIF-8, although AgNPs have (111), (200), (220), and (311) crystal planes, sharp diffraction peaks of (011), (112), and (222) crystal planes of ZIF-8 are also observed at 2θ = 5–20° (inset image of Figure 2J), indicating good crystallinity of ZIF-8 encapsulated on AgNPs@Si. In N_2_ adsorption-release experiments, the N_2_ adsorption of Si@AgNPs@ZIF-8 was slightly lower than that of pure ZIF-8 (Figure 2K). The BET specific surface areas of ZIF-8 and Si@AgNPs@ZIF-8 were calculated to be 1209.16 m^2^ g^−1^ and 977.33 m^2^ g^−1^, respectively. These characterization results demonstrated that Si@AgNPs@ZIF-8 was successfully synthesized by microfluidics.

### Quantitative SERS detection of VOCs

**Figure 3:**
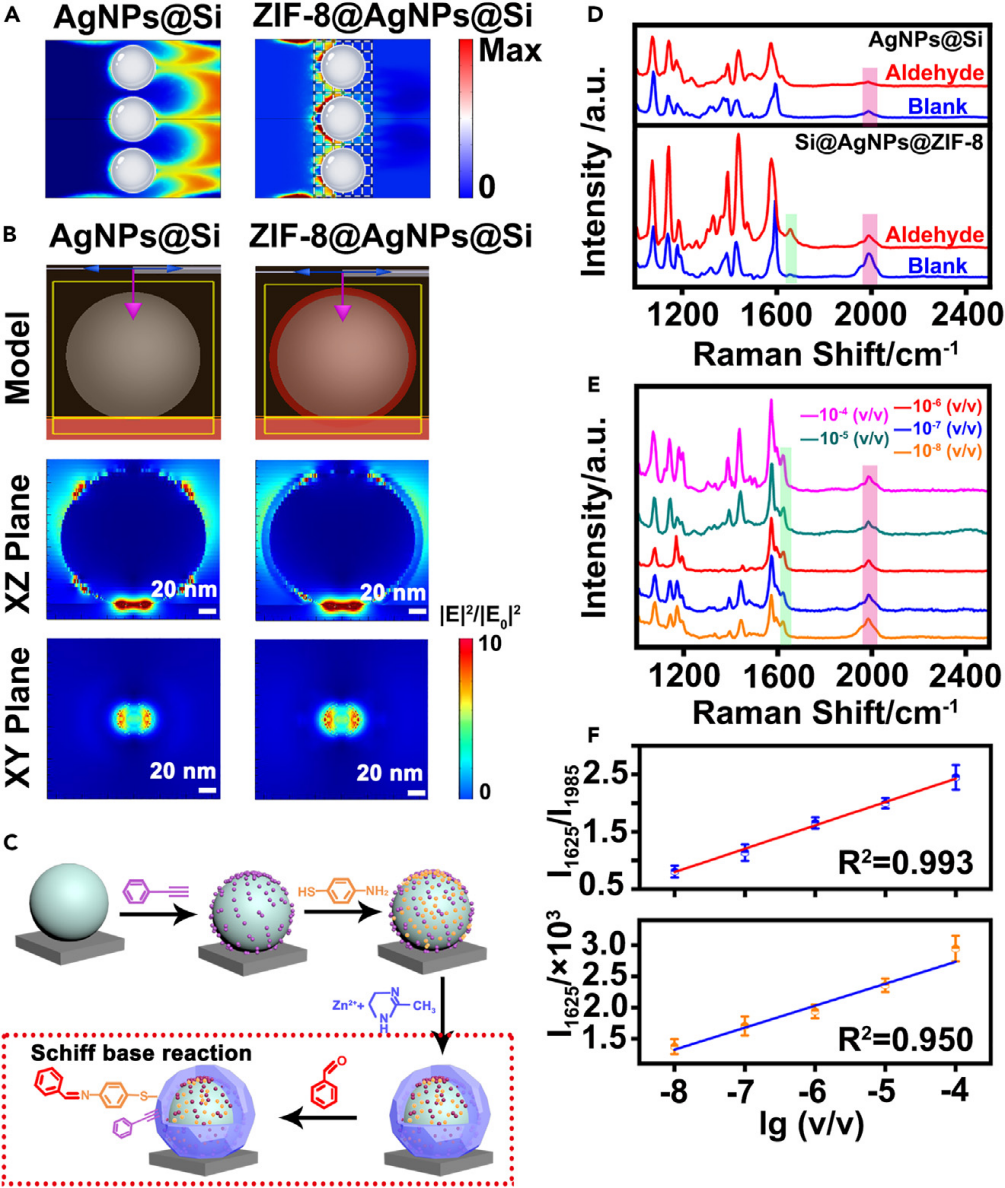
Quantitative detection of benzaldehyde by the developed SERS chip (A) Simulated concentration of gas diffusing through AgNPs@Si and Si@AgNPs@ZIF-8. Flow rate = 0.001 mol/m^2^·s, time point = 120 s. (B) FDTD simulation of the EM-field intensity (|E|^2^/|E0|^2^) distribution of AgNPs@Si and Si@AgNPs@ZIF-8. |E|^2^ is the scattered EM-field intensity, and |E_0_|^2^ is the incident EM-field intensity. The radius of the AgNPs is 82 nm, the ZIF-8 thickness is 10 nm, and the incident laser of the plane wave (purple arrow) in the (−) z direction is 514 nm. (C) Schematic illustration of phenylacetylene (IS) and 4-ATP functionalized Si@AgNPs@ZIF-8 for quantitative detection of benzaldehyde based on the Schiff base reaction and IS calibration. (D) SERS spectra collected from functionalized AgNPs@Si before and after reaction with benzaldehyde (top panel) and SERS spectra collected from functionalized Si@AgNPs@ZIF-8 before and after reaction with benzaldehyde (bottom panel). (E and F) (E) SERS spectra collected from functionalized Si@AgNPs@ZIF-8 in the detection of benzaldehyde with different concentrations ranging from 10^−8^ to 10^−4^ (v/v) and (F) corresponding linear fitting of ratiometric Raman intensities (I_1625_/I_1985_) with logarithmic benzaldehyde concentrations (top panel) and the corresponding linear fitting of Raman signals (I_1625_) with logarithmic benzaldehyde concentrations (bottom). I_1625_ and I_1985_ stand for Raman intensity at 1625 cm^−1^ and 1985 cm^−1^, respectively. The error bars show the standard deviation determined from three independent assays. Excitation wavelength = 633 nm, acquisition time = 1 s, laser power = 20 mW.

To primarily explore the ability of Si@AgNPs@ZIF-8 to capture VOC molecules, we performed simulations of gaseous molecules diffusing through the fabricated nanostructures by COMSOL Multiphysics. Briefly, the concentration of gaseous molecules passing through Si@AgNPs@ZIF-8 and AgNPs@Si at any given time point was calculated by Fick’s second law (Reading et al., 2006):

(Equation 1) $$\partial c/\partial t=\text{Da}((\partial^{2}c)/\left(\partial x^{2}\right))$$

where *c* is the gas concentration, *t* is time, *Da* is the binary molecular diffusion coefficient in air, and x is the distance along the axis of flow. As shown in Figure 3A, the gaseous analytes were still retained in the Si@AgNPs@ZIF-8 array with a high concentration at 120 s (right panel), while they almost passed through the AgNPs@Si array under identical conditions (left panel). The simulation results proved that VOC molecules could be effectively confined in Si@AgNPs@ZIF-8, increasing the adsorption of VOCs onto substrates.

The SERS enhancement of Si@AgNPs@ZIF-8 was tested. The electromagnetic (EM)-field distribution of substrates was first simulated by the finite-difference-time-domain (FDTD) method. As presented in Figure 3B, relatively strong EM fields were observed around silver nanoparticles and in the gap between silver nanoparticles and silicon surfaces in both AgNPs@Si and Si@AgNPs@ZIF-8. Of note, the intensity of the EM field in the gap between the silver nanoparticle and silicon surface in Si@AgNPs@ZIF-8 was slightly stronger (e.g., 2-fold enhancement) than that in AgNPs@Si. As previously reported, the EM field decayed exponentially with distance from the substrate.^42^ In this case, the penetration depth (Z) of the EM field can be determined by Equation 2:

(Equation 2) $$Z=\lambda /2\pi \left[\left(\varepsilon_{\text{medium}}^{\prime }-\varepsilon_{\text{Ag}}^{\prime }\right)/\left({\varepsilon_{\text{Ag}}^{\prime }}^{2}\right)\right]/2$$

where $\varepsilon_{medium}^{\prime }$ is the dielectric constant of ZIF-8 or air and $\varepsilon_{Ag}^{\prime }$ is the dielectric constant of silver nanoparticles. At 633 nm, $\varepsilon_{ZIF-8}^{\prime }$ = 1.34 and $\varepsilon_{air}^{\prime }$ = 1.0003; therefore, ZSi@AgNPs@ZIF-8 > ZAgNPs@Si. As a result, the ZIF-8 shell with ca. A 10-nm thickness guaranteed a distinct plasmonic enhancement. We compared the Raman signal intensity at 1625 cm^−1^ in the presence of 100 ppb benzaldehyde collected from the SERS substrates with different thickness of MOF shell (e.g., 10 nm, 50 nm and 100 nm). As shown in Figure S4, the strongest signal intensity was obtained when the shell layer thickness was 10 nm. In line with theoretical discussions, the optimum thickness is 10 nm.

To realize quantitative detection, phenylacetylene and *p*-aminothiophenol (4-ATP) molecules were pumped into the microchannel at a flow rate of 6 μL/min before the formation of the ZIF-8 shell. In particular, phenylethynyl as an internal standard (IS) probe is modified on the AgNP@Si surface via a covalent bond between the alkyne group of phenylethynyl and AgNPs, while 4-ATP as a signal probe is modified on the AgNP@Si surface via a covalent bond between the sulfhydryl group of 4-ATP and AgNPs. Theoretically, aldehyde molecules (e.g., benzaldehyde) were specifically captured onto Si@AgNPs@ZIF-8 through a Schiff base reaction with 4-ATP. The aldehyde group (-CHO) of benzaldehyde reacted with the amino group of 4-ATP to form a new C=N bond (Figure 3C).

Benzaldehyde was selected to validate the SERS quantification ability of Si@AgNPs@ZIF-8. As revealed in Figure 3D, first, much stronger Raman signals of 4-ATP were observed in functionalized Si@AgNPs@ZIF-8, with approximately 2.3-fold higher enhancement than that of functionalized AgNPs@Si. The distinct SERS enhancement observed in Si@AgNPs@ZIF-8 was in agreement with the aforementioned FDTD simulation results. Second, a new Raman peak at 1625 cm^−1^ (assigned to the C=N stretching mode of imine) appeared not in functionalized AgNPs@Si (top panel) but in functionalized Si@AgNPs@ZIF-8 (down panel). Of note, the Raman peak at 1985 cm^−1^ located in the Raman silent region (assigned to the alkynyl group of phenylacetylene) was employed to calibrate the SERS signals of 4-ATP. Specifically, the intensity of the 1625 cm^−1^ peaks gradually increased with the increase of benzaldehyde concentration (from 10^−8^ to 10^−4^ v/v), displaying concentration-dependent behavior (Figure 3E). However, there was a poor linearity between the logarithmic concentration of benzaldehyde and peak intensity at 1625 cm^−1^ with a low correlation coefficient *R*^2^ of 0.95 (Figure 3F). By calibration with IS signals at 1985 cm^−1^, a good linearity between the logarithmic concentration of benzaldehyde and the ratiometric signal (I_1625_/I_1985_) was achieved with a high *R*^2^ of 0.993. The limit of detection (LOD) was down to 8 × 10^−9^ (ppb level) by setting the signal-to-noise ratio to 3:1. In addition, this SERS chip showed a good selectivity to benzaldehyde against H_2_O, acid, acetone, alcohol, and benzene, exhibiting the feasibility of anti-interference for the detection of complex samples (Figure S5). At the same time, the distribution of benzaldehyde on the substrate is quite homogeneous, the relative standard deviation (RSD) is 14.2% (Figure S6).

### Discrimination of various VOCs at the ppb level

**Figure 4:**
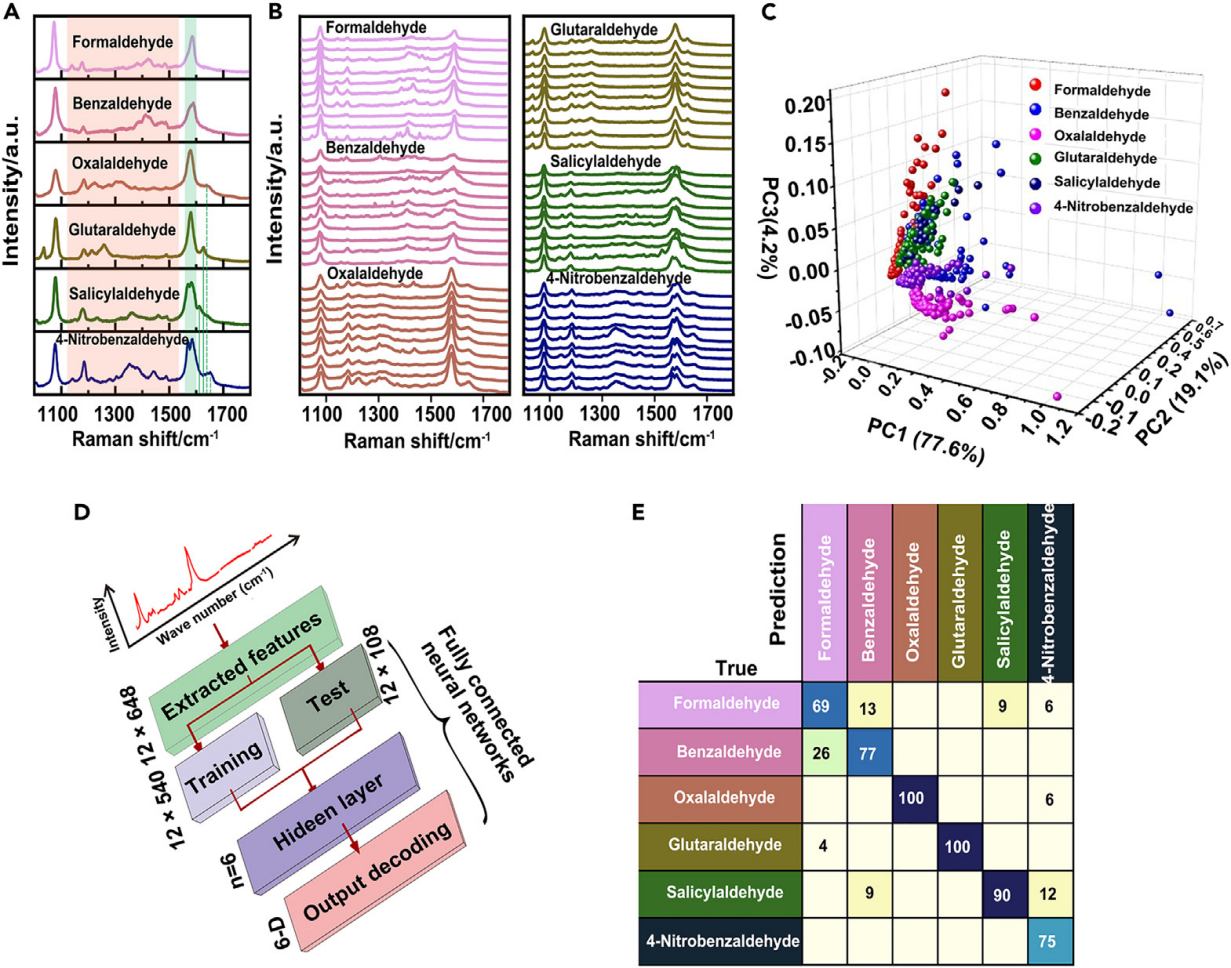
Discrimination of ppb-level aldehydes by using SERS and DNN (A) Representative SERS spectra collected from functionalized Si@AgNPs@ZIF-8 in the detection of various aldehydes (formaldehyde, benzaldehyde, oxalaldehyde, glutaraldehyde, salicylaldehyde and 4-nitrobenzaldehyde) with the same concentration of 100 ppb. (B) Representative examples of the SERS database. (C) PCA of SERS spectra from the detection of six types of aldehydes. (D) Architecture of the customized fully connected neural network. (E) Confusion matrix for six aldehyde classes. Entries along the diagonal represent the prediction accuracies for each aldehyde. Percentages below 1% are not shown.

To discriminate different VOC analytes, we selected six different gaseous aldehydes, including formaldehyde, benzaldehyde, oxalaldehyde, glutaraldehyde, salicylaldehyde and 4-nitrobenzaldehyde (Table S1), with the same concentration of 100 ppb to react with Si@AgNPs@ZIF-8. Figure 4A showed the characteristic Raman spectra of different aldehydes after reaction with the SERS chip. Typically, the peaks of 1632 cm^−1^ in oxalaldehyde, 1627 cm^−1^ in glutaraldehyde, 1610 cm^−1^ in salicylaldehyde and 1652 cm^−1^ in 4-nitrobenzaldehyde (green lines) were assigned to the C=N stretching mode of aldehydes; the peaks within 1569–1589 cm^−1^ among different aldehydes appeared with different shifts, which were assigned to the C-C stretching mode (green shadow). Of note, two split peaks within this range were observed in salicylaldehyde and 4-nitrobenzaldehyde. Other peaks appearing in the range of 1100–1500 cm^−1^ were assigned to C-H deformation, C-C stretching and C-S stretching (orange shadow). The detailed assignments of the Raman spectra were listed in Table S2. Despite these certain spectral features in the single representative spectra of each target, numerous SERS spectra were collected and presented variable spectral differences mainly in the range of 1100–1500 cm^−1^ (Figure 4B). SERS spectra datasets containing intensities-variable certain peaks and erratic miscellaneous peaks were further discriminated by computational algorithms.

To distinguish these aldehydes, we tried to use conventional principal component analysis (PCA) to arrange the SERS spectra into groups based on the degree of resemblance.^16^ However, the clusters of six aldehydes overlapped with each other, as shown in the three-dimensional PCA score plot (Figure 4C). This result suggested that PCA hardly separated these aldehydes at the 100 ppb level in this case. Alternatively, deep learning has been applied with tremendous success to a broad range of spectral data processing.^38^^,^^43^^,^^44^^,^^45^ Inspired by these elegant works, we built a fully connected DNN containing one hidden layer with six neurons (Figure 4D). To train this neural network with robust data, each original spectrum (a total of 648 data points) was divided into 12 sections according to the range of Raman shift, and the shifting position of the highest peak in each range was extracted as 12 input parameters (12 × 648). The DNN model was then trained on the training dataset (12 × 540) and validated on a separate test dataset (12 × 108). After training, the algorithm exported the confusion matrix of the test dataset, as shown in Figure 4E. On this 6-class classification task, an accuracy of ∼84.2% was finally achieved. The assessment was further evaluated by receiver operating characteristic (ROC) curves. Areas under the ROC curves of formaldehyde, benzaldehyde, oxalaldehyde, glutaraldehyde, salicylaldehyde and 4-nitrobenzaldehyde were 0.95, 0.96, 1.00, 1.00, 0.92 and 0.99, respectively, while areas under the macroaverage and microaverage ROC curves were both 0.97 (Figure S7). These results suggested that Si@AgNPs@ZIF-8 assisted with DNN presented good performance in distinguishing different aldehydes with relatively low concentrations.

### Discrimination of various VOCs at the ppt level

**Figure 5:**
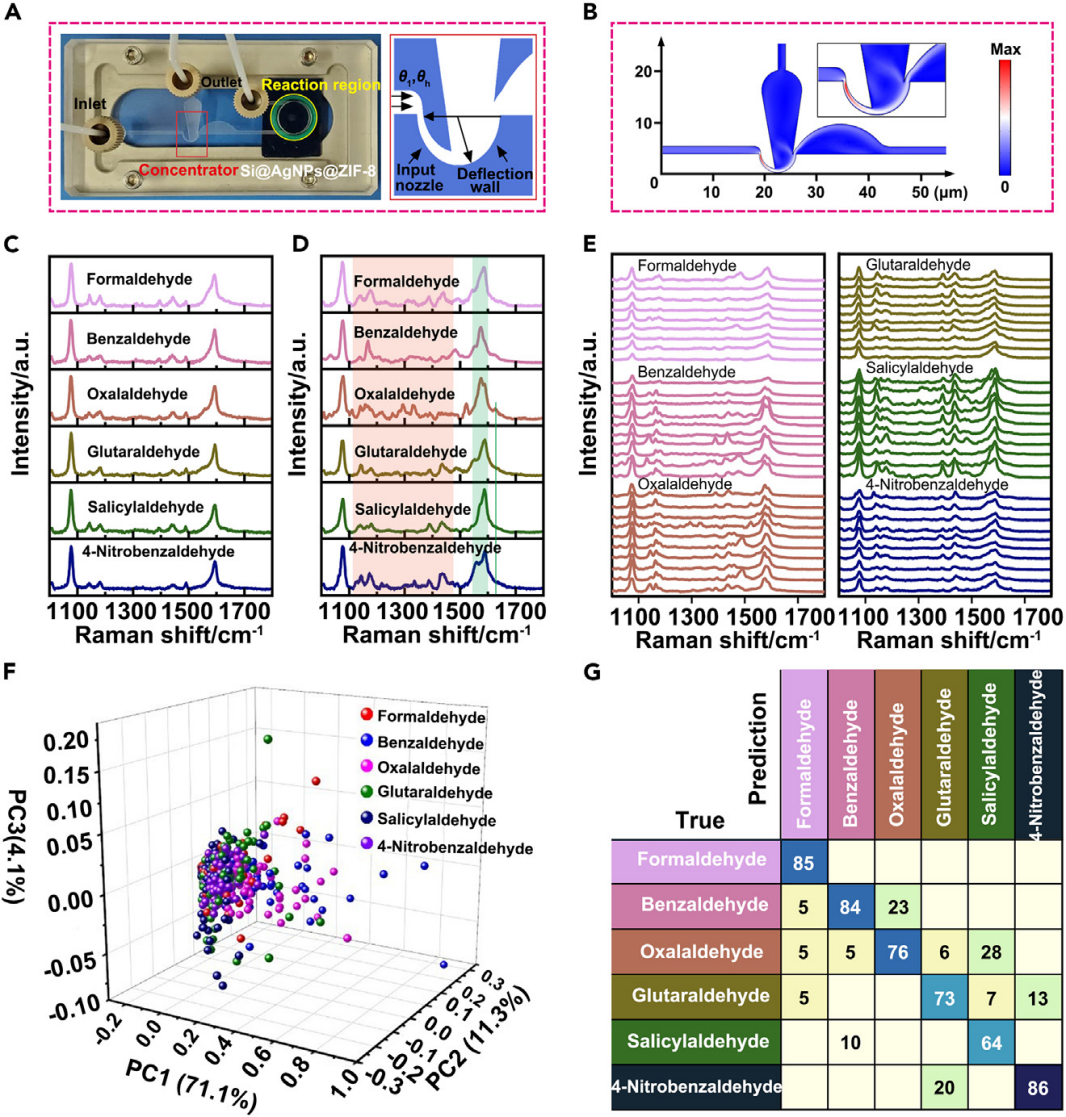
Discrimination of ppt-level aldehydes using SERS-active microfluidic device combined with deep learning (A) Photograph of the microfluidic concentrator integrated with the SERS detection chip and the detailed design of the concentrator pattern (right frame). (B–D) (B) Simulation of the speed of N_2_ in air passing through the microfluidic concentrator. Representative SERS spectra for the detection of 100 ppt aldehydes (formaldehyde, benzaldehyde, oxalaldehyde, glutaraldehyde, salicylaldehyde and 4-nitrobenzaldehyde) in a non-concentrating (SERS) (C) or concentrating (SERS-active microfluidic) system (D). (E) Corresponding examples of the SERS database collected from the detection of 100 ppt aldehydes in the SERS-active microfluidic system. (F) PCA of SERS spectra from the detection of six types of aldehydes. (G) Confusion matrix for six aldehyde classes. Entries along the diagonal represent the prediction accuracies for each aldehyde. Percentages below 1% are not shown.

Solid SERS substrates were in principle well compatible with microfluidic devices. To achieve higher sensitivity, a microfluidic concentrator was designed and fabricated to couple with Si@AgNPs@ZIF-8. The designed microfluidic device could reduce gas consumption and divide gaseous mixtures into light fractions and heavy fractions (such as VOCs) based on the separation nozzle mechanism. As illustrated in Figure 5A, the concentrator consisted of an input nozzle, a deflection wall and a skimmer. The Si@AgNPs@ZIF-8 was sandwiched into the microfluidic device in the SERS-active region. In such an online concentrator, the gaseous mixture would be accelerated by the nozzle and then deflected by the curved wall. Of note, a radical pressure gradient would be generated by the centripetal acceleration, which was related to the turning flow. The as-generated pressure gradient would lead to the differential diffusion of the light fraction and heavy fraction in directions perpendicular to the streamlines. As a consequence, the heavy fraction would be concentrated at the periphery of the flow field and mechanically separated by a skimmer in the curved channel. Theoretically, the separation factor (*A*) was employed to describe the concentration change between the light and heavy fractions. Therefore, a successful preconcentrator could maximize the separation factor. The separation factor (*A*) was closely associated with the partial cuts (*θ*_*i*_). For a specific gas mixture, its partial cut was defined as the percentage of its throughput in the separation element, which was determined by the geometric parameters of the concentrator, such as the throat width (*a*) and exit width (*w*) of the nozzle, the radius of the deflection wall (r) and the skimmer distance (*f*), as illustrated in the right frame of Figure 5A. To achieve the maximum separation factor, a series of systematic optimizations were performed. Ultimately, the throat width of the nozzle (*a*) was set as 200 μm, the exit width of the nozzle (*w*) was set as 500 μm, the radius of the deflection wall (*r*) was set as 2.4 mm, and the skimmer distance (*f*) was set as 400 μm.

Afterward, we simulated the speed distribution of gas flow in the microfluidic concentrator by COMSOL Multiphysics software. A gas mixture of SF_6_ diluted in N_2_ was selected as the modeling species. As shown in Figure 5B, the gas speed reached a maximum value in the first corner 90° downwards. Along the deflection wall, the gas flow spiked into two fractions, and one of the fractions flowed to the second corner 90° downwards with a comparably high speed. The heavier gas with higher speed was mechanically separated into the downstream chamber for the following reaction with the SERS chip. As a consequence, compared with the initial diluted SF_6_, five-fold enrichment of SF_6_ was achieved in the reaction region with a sharply short response time of 0.01 ms, and such performance was further confirmed by practical measurement with GC‒MS.

Six gaseous aldehydes, with an input concentration of 100 ppt in a total volume of 1 mL, were detected in both non-concentrating (SERS) and concentrating (SERS-active microfluidic) systems. In the nonconcentrated system (Figure 5C), peaks at 1141 cm^−1^ (*δ*(C-H)), 1179 cm^−1^ (*δ*(C-H)) and 1593 cm^−1^ (*υ*(C-C)) originating from 4-ATP were observed in the other aldehyde groups, and the positions of these peaks were not changed. In addition, no typical peaks assigned to the C=N stretching mode were observed. These findings suggested that there were no significant differences in SERS spectra among diverse aldehydes of 100 ppt recorded in the non-concentrating system. However, in the concentrating system (Figure 5D), specific Raman peaks at 1585 cm^−1^ (*υ*(C-C)) in formaldehyde, Raman shifts at 1574 cm^−1^ (*υ*(C-C)) in benzaldehyde, Raman peaks at 1575 cm^−1^ (*υ*(C-C)) and 1625 cm^−1^ (*υ*(C=N)) in oxalaldehyde, Raman peaks at 1588 cm^−1^ (*υ*(C-C)) in glutaraldehyde, Raman shifts at 1587 cm^−1^ (*υ*(C-C)) in salicylaldehyde, and Raman shifts at 1558 cm^−1^ and 1588 cm^−1^ (*υ*(C-C)) in 4-nitrobenzaldehyde can be observed (Table S3). These Raman shifts and other erratic miscellaneous peaks were extracted as the main features for the discrimination of each target by neural networks, as described in the aforementioned detection of ppb-level VOCs. As such, these SERS spectra were collected as datasets for automated computational discrimination (Figure 5E). Similar to ppb-level detection, the result of PCA showed a poor degree of separation according to different aldehyde classes (Figure 5F). In contrast, the DNN effectively discriminated these aldehydes. After training, an accuracy of ∼80.9% was achieved in the assessment of the test dataset (Figure 5G), and the areas under the ROC curves of the six gaseous targets were 0.92, 0.97, 0.93, 0.90, 0.94 and 1.00 (Figure S8). It was worth noting that the overall accuracy in this case was lower than that in the case of ppb-level detection because 4-ATP on the surface of Si@AgNPs@ZIF-8 could react with fewer aldehyde molecules, making more chances to generate spectra with analogous SERS spectra. We compared the effect of 100 ppt benzaldehyde with the Si@AgNPs@ZIF-8 with and without the preconcentration. Much stronger Raman signal at 1625 cm^−1^ was observed in the chip with preconcentration, with approximately 4.32-fold higher enhancement than that of the chip without preconcentration, indicating the significant role of preconcentration. (Figure S9).

## Discussion

We first discuss the difficulties faced by current gas sensors in the analysis of sensing, because current gas sensors are difficult to distinguish ppt level trace VOCs, herein we present an integrated platform for simultaneously enabling rapid preconcentration, reliable SERS detection and automatic identification of trace aldehydes at the ppt level. To improve the detection sensitivity, we added a nozzle-type microfluidic concentrator to the device, which can enrich the noble gas analytes by a factor of 5 in 0.01 ms. An integrated silicon-based SERS device, composed of silver nanoparticles with zeolitic imidazolate framework-8 coating that were produced *in situ* on a silicon wafer, is then used to trap and detect the enriched gas. A fully connected DNN is created to extract weak features from the spectrum dataset and distinguish between classes of volatile organic molecule after SERS measurement. We show that utilizing this platform, six different types of gaseous aldehydes at 100 ppt can be identified with an accuracy of about 80.9%.

### Conclusions

In this study, we presented the first demonstration of the discrimination of various VOCs at the ppt level by using a novel SERS-active microfluidic device combined with deep learning. First, we synthesized AgNP@Si substrates coated with ca. 10 nm-thick ZIF-8 layer in a developed microfluidic system, effectively enhancing the adsorption of VOCs. We further employed a microfluidic separation nozzle as an auxiliary medium for the preconcentration of VOCs before SERS measurements. We demonstrated that such a concentrator achieved 5-fold enrichment of gas in 0.01 ms. To discriminate SERS spectra collected from VOCs at the ppt level, we built a fully connected DNN containing one hidden layer with six neurons, establishing a unified standard SERS data processing method. On this basis, six different kinds of aldehydes with low concentrations down to 100 ppt can be readily discriminated with a high accuracy rate up to 80.9%. Such a hypersensitive sensing strategy has the potential to obtain full and accurate VOC information in breath analysis, laying a foundation for precise diagnosis at an early stage.

## STAR★Methods

### Key resources table

**Table:** 

REAGENT or RESOURCE	SOURCE	IDENTIFIER
**Chemicals, peptides, and recombinant proteins**		

Acetone	Sinopharm Group Co., Ltd.	CAS No. 67-64-1
Hydrogen peroxide	Sinopharm Group Co., Ltd.	CAS No. 7722-84-1
Sulfuric acid	Sinopharm Group Co., Ltd.	CAS No. 8014-95-7
Hydrofluoric acid	Sinopharm Group Co., Ltd.	CAS No. 7664-39-3
AgNO_3_	Sinopharm Group Co., Ltd.	CAS No. 7761-88-8
Phenylacetylene	Sinopharm Group Co., Ltd.	CAS No. 536-74-3
Aminothiophenol	Sinopharm Group Co., Ltd.	CAS No. 1193-02-8
Zinc nitrate hexahydrate	Sinopharm Group Co., Ltd.	CAS No. 10196-18-6
2-methylimidazole	Sinopharm Group Co., Ltd.	CAS No. 693-98-1
Methanol	Sinopharm Group Co., Ltd.	CAS No. 67-56-1
Ethanol	Sinopharm Group Co., Ltd.	CAS No. 64-17-5
Formaldehyde	Sinopharm Group Co., Ltd.	CAS No. 50-00-0
Benzaldehyde	Sinopharm Group Co., Ltd.	CAS No. 00-52-7
Oxalaldehyde	Sinopharm Group Co., Ltd.	CAS No. 107-22-2
Glutaraldehyde	Sinopharm Group Co., Ltd.	CAS No. 111-30-8
Salicylaldehyde	Sinopharm Group Co., Ltd.	CAS No. 90-02-8
4-nitrobenzaldehyde	Sinopharm Group Co., Ltd.	CAS No. 555-16-8

**Software and algorithms**		

Origin	www.originlab.com	Origin 2018
COMSOL	www.comsol.com	COMSOL Multiphysics software ver. 5.3
Finite-difference time domain (FDTD) simulation	www.lumerical.com	Lumerical® FDTD Solutions 8.5
Deep neural networks	https://opensource.google/projects/tensorflow	Google open source project TensorFlow (release 2.1)
Principal component analysis	https://pypi.org/project/scikit-learn/	sklearn toolkits (python 3.7)

**Other**		

P-type silicon wafers	Hefei Kejing Ltd.	NA
Scanning electron microscopy	FEI Quanta 200F	NA
Atomic force microscope	Vecco Corp.	NA
X-ray diffraction	ANalytical, Empyrean, X-ray diffractometer	NA
Nitrogen adsorption-desorption isotherms	ASAP2020 (Micromeritics Industrument Corp.)	NA
Raman spectra	Horiba Jobin Yvon, HR800	NA

### Resource availability

#### Lead contact

Further information and requests for resources and reagents should be directed to and will be fulfilled by the lead contact, Prof. Houyu Wang (houyuwang@suda.edu.cn).

#### Materials availability

This work has not released any new products. This study did not generate new unique reagents.

### Method details

#### Fabrication of SERS chip

To synthesize silver nanoparticles modified silicon wafer (AgNPs@Si), solutions of 1.25 mM silver nitrate (10% HF) were pumped into a microfluidic device at 6 μL/min for a 12 min reaction, and Milli-Q water was then pumped into the device to stop the reaction and wash the product. To functionalize AgNPs@Si, 10^-5^ M phenylacetylene and 10^-5^ M 4-aminothiophenol (4-ATP) in EtOH were subsequently pumped into the microfluidic device at 6 μL/min for 1 h of modification, and MeOH was then pumped into the device to wash the modified product. To form the ZIF-8 shell, 0.298 g Zn(NO_3_)_2_·6H_2_O was dissolved in 20 mL of MeOH, and 0.164 g 2-methylimidazole was dissolved in 10 mL of MeOH. The two solutions were mixed and then pumped into the microfluidic device at 6 μL/min for a 1 h reaction, and the product chip was finally washed with MeOH and DI water three times and dried overnight. A nitrogen interval (∼ 100 mm long in the catheter) was introduced using an injector to separate different solutions between steps all the time. All pumping operations were controlled by a syringe pump (Suzhou Wenhao Microfluidic Technology, WH-SP-02-type).

#### Fabrication of SERS-active microfluidic device

The polymethyl methacrylate (PMMA) layer of the SERS-active microfluidic device had two connected functional units: one was the microfluidic concentrator (separation nozzle region), and the other was the SERS detection chamber (SERS-active region). In the microfluidic concentrator unit, there was an inlet for gas injection and an outlet for discharging light fraction gas (1 mm in diameter). The main structure of the curved converging-diverging nozzle was formed by the deflection wall and the inner wall. In the SERS detection chamber, the Si@AgNPs@ZIF-8 substrate was coupled to the chamber (5 mm in diameter) through a sealing ring, followed by an outlet (1 mm in diameter) for discharging the gaseous species. The PMMA layer was integrated and fixed with the SERS chip and aluminum alloy bracket by the clamp.

#### Gas sensing

For non-concentrating detection, the SERS substrates were placed in a Teflon chamber with inlet and outlet pipelines, and aldehyde VOCs with different concentrations were pumped into the chamber by a mass flow controller (5850E, Brooks Instruments) with a flow rate of 100 mL/min. The whole sensing system was set at a controlled temperature to maintain a stable vapor pressure. The typical reaction time between VOC analytes and SERS substrates was 20 min. For concentrating detection, aldehyde VOCs were pumped into the microfluidic device with a flow rate of 1 mL/min. The reaction time was set as 1 min. Unless otherwise noted, other conditions were the same as for non-concentrating detection. The VOC concentrations were calibrated with a commercial VOC sensor (Honeywell, ToxiRAE Pro PID).

#### Simulation of the finite-difference time domain (FDTD)

The simulations were calculated by Lumerical® FDTD Solutions 8.5. The models of the AgNPs@Si and Si@AgNPs@ZIF-8 were constructed according to the SEM images. The electric permittivities of silicon and silver were set from the material library of FDTD Solutions. The n/k index of ZIF-8 was measured with an ellipsometer (J. A. Woollam Co., Inc.). The diameter of the AgNPs was set as 168 nm, the thickness of the ZIF-8 shell was set as 10 nm, and the dimensions of the silicon substrate were set as 1 μm (x) × 1 μm (y) × 0.5 μm (z). The FDTD simulation volume was 1 μm (x) by 1 μm (y) by 0.7 μm (z) with perfectly matched layer boundaries along the x-, y- and z-axes. The minimum mesh step was set as 0.5 nm. A plane wave of 514 nm propagating along the -z direction was used as the excitation source, and the polarization of the laser was along the -x direction.

#### DNN architecture and training

The DNN architecture consisted of three layers: input layer (12 neurons), hidden layer (6 neurons) and output layer (6 neurons). The hidden layer receives signals from the input layer and delivers signals to the output layer. A nonlinear softmax activation function was employed to transfer the signals. To fit the DNN architecture, 12 input parameters were extracted from the highest peak shifting of 12 separated Raman shifting regions (1000-2300 cm^-1^ separated by 1030, 1152, 1174, 1195, 1280, 1350, 1410, 1450, 1522, 1545, 1800 cm^-1^) of each spectrum in the original dataset, and the output was defined as a 6-dimensional vector by one hot encoding. The transformed dataset was randomly separated into a training dataset (12×540,6-D×540) for DNN training and a test dataset (12×108,6-D×108) for validation. All data processing, network building and training were implemented in Google open source project TensorFlow (release 2.1). In both ppb-level and ppt-level detection, the original dataset consisted of 648 SERS spectra in which 108 spectra each for the 6 different aldehydes were collected from 4 independent measurements. PCA was performed using original SERS intensity data for Raman shifting of 1000-2300 cm^-1^ in sklearn toolkits (python 3.7).

## Data Availability

• Data reported in this paper will be shared by the lead contact upon request. • This paper does not report original code. • Any additional information required to reanalyze the data reported in this paper is available from the lead contact upon request.
