# Supplementary material for: Ultrasensitive discrimination of volatile organic compounds using a microfluidic silicon SERS artificial intelligence chip
Source: iScience. 2023 Sep 2;26(10):107821. doi: 10.1016/j.isci.2023.107821 (PMC10507157; doi:10.1016/j.isci.2023.107821)
Supplement: Document S1. Figures S1–S9 and Tables S1–S3 [file mmc1.pdf]

iScience, Volume *26*

Supplemental information

Ultrasensitive discrimination of volatile organic compounds using a microfluidic silicon SERS artificial intelligence chip

Haiting Cao, Huayi Shi, Jie Tang, Yanan Xu, Yufan Ling, Xing Lu, Yang Yang, Xiaojie Zhang, and Houyu Wang

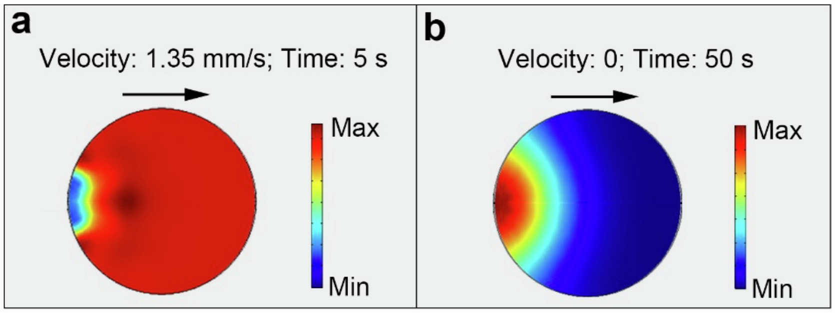

Figure S1. Numerical simulation of ion distribution in different systems. Simulation result of the panel 0.01 mm above the silicon substrate in the microfluidic GD (a) and bulk GD (b). **Related to Figure 2.**

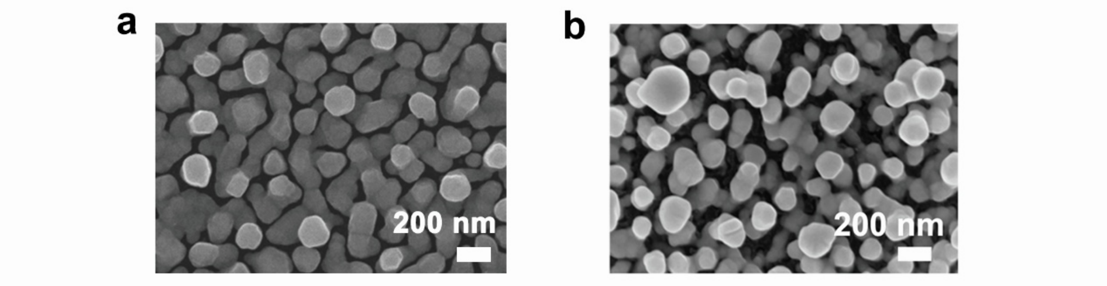

Figure S2. SEM images of AgNPs@Si. SEM images of AgNPs@Si synthesized by microfluidic GD (a) and bulk GD (b). **Related to Figure 2.**

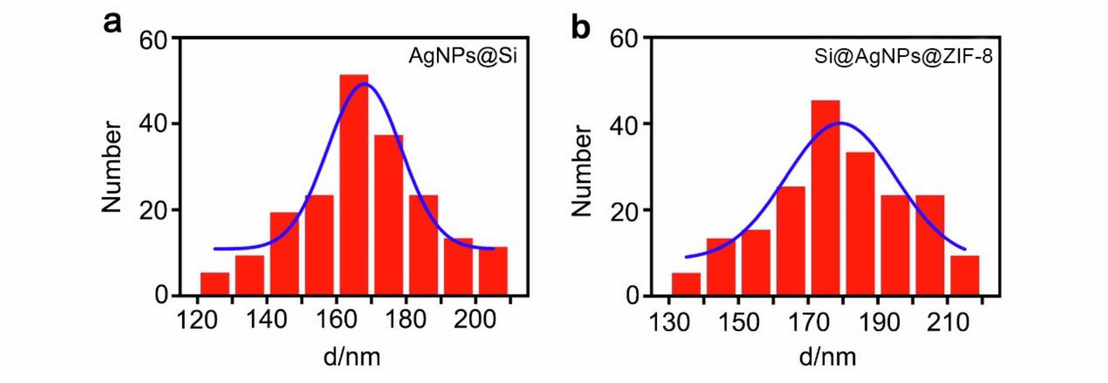

Figure S3. Statistical analysis of SERS substrates synthesized by microfluidic methods. Size distributions of AgNPs@Si (a) and Si@AgNPs@ZIF-8 (b). **Related to Figure 2.**

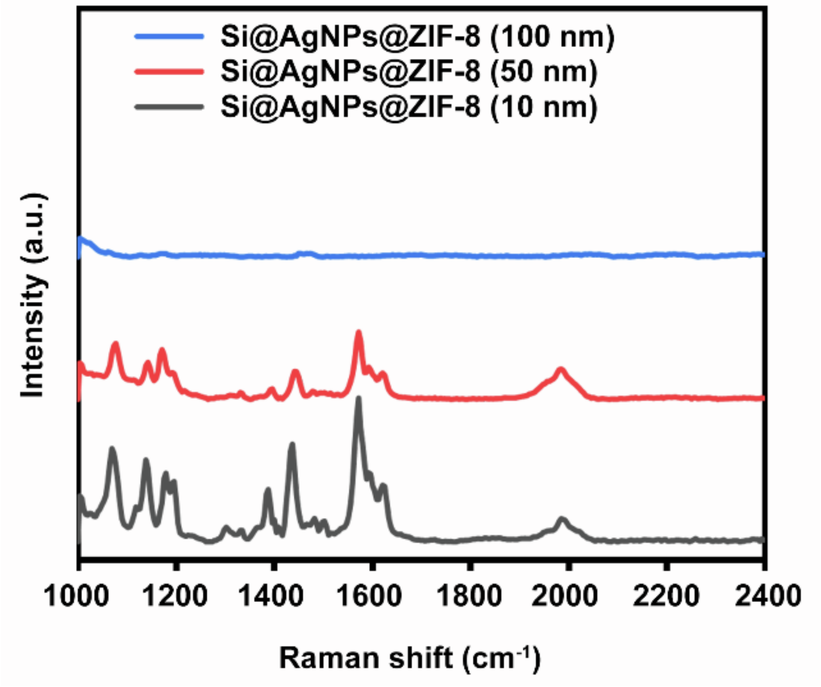

Figure S4. Comparison of Raman signals of the SERS substrates with different thicknesses of MOF shell. **Related to Figure 3.**

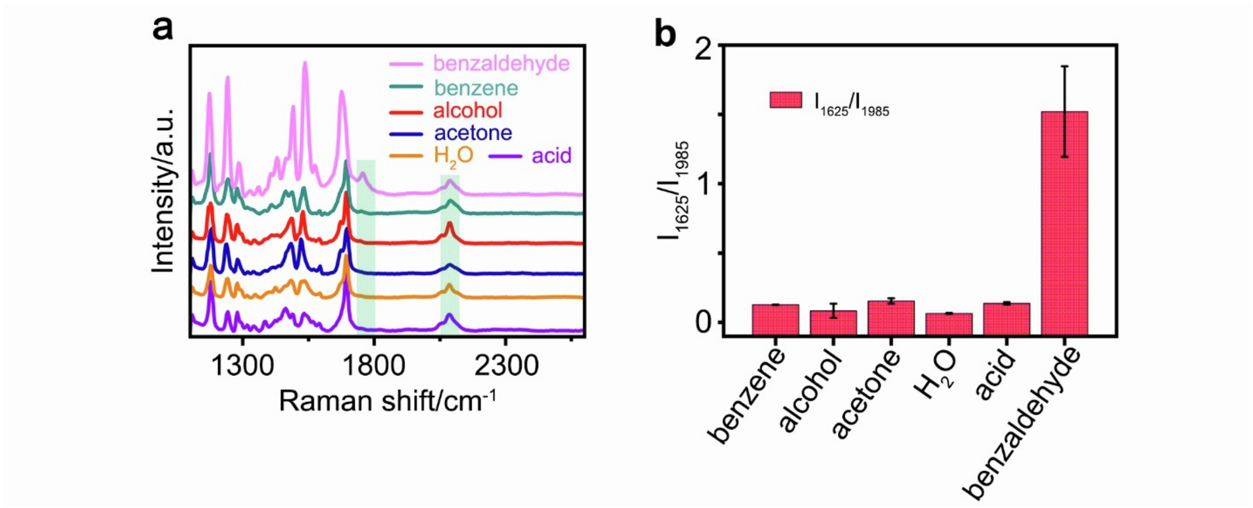

Figure S5. Detection specificity of the 4-ATP-modified Si@AgNPs@ZIF-8 SERS sensor. (a) SERS spectra and (b) the corresponding ratiometric intensities of I_{1625}/I_{1985} in the detection of benzaldehyde, benzene, alcohol, acetone, H_2O and acid. Error bars present the standard deviation obtained from three independent measurements. **Related to Figure 3.**

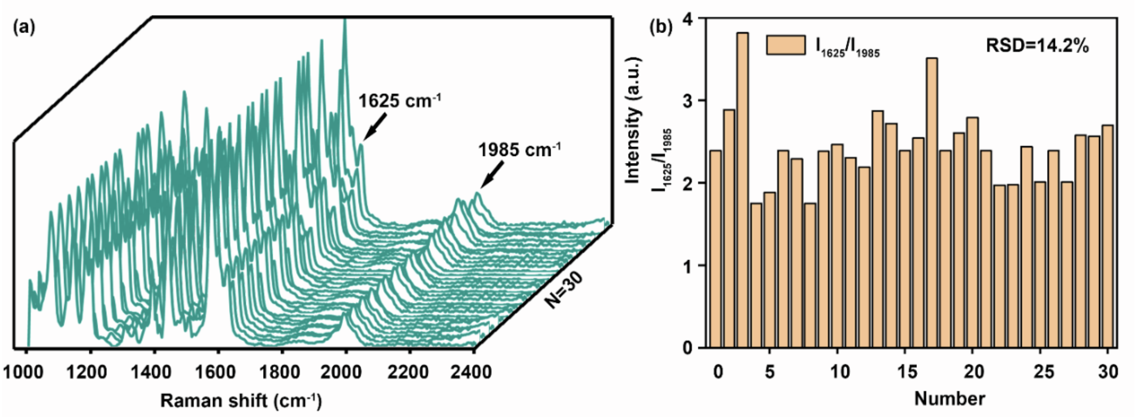

Figure S6. The distribution homogeneous of benzaldehyde molecules on the Si@AgNPs@ZIF-8 substrate. (a) SERS mapping spectra of the Si@AgNPs@ZIF-8 substrate and (b) corresponding ratiometric values collected from 30 spots over a defined area of 30 × 30 μm^2. Excitation wavelength = 633 nm; acquisition time = 1 sec, laser power = 20 mW. **Related to Figure 3.**

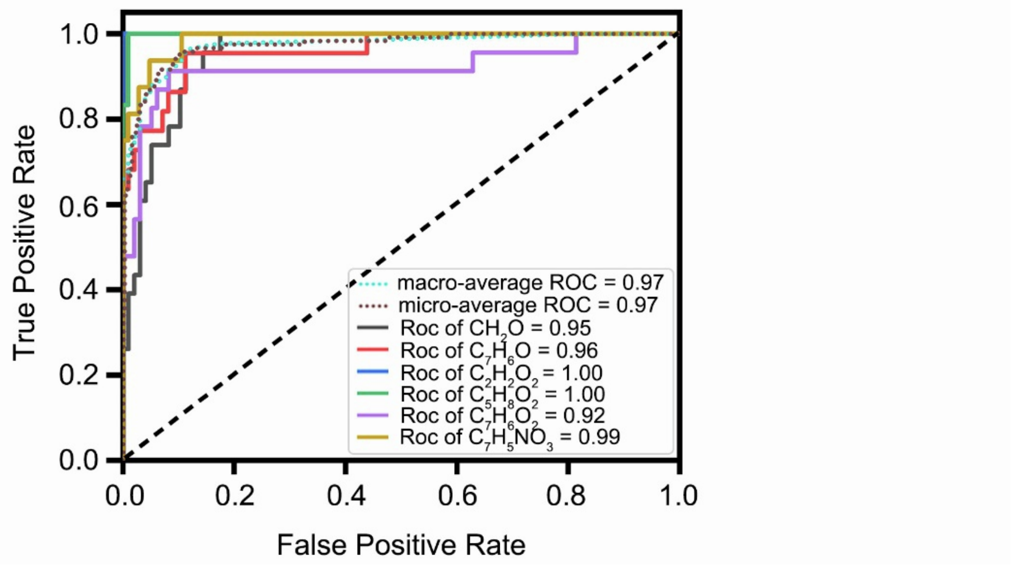

Figure S7. ROC curves of different aldehyde targets (ppb-level). **Related to Figure 4.**

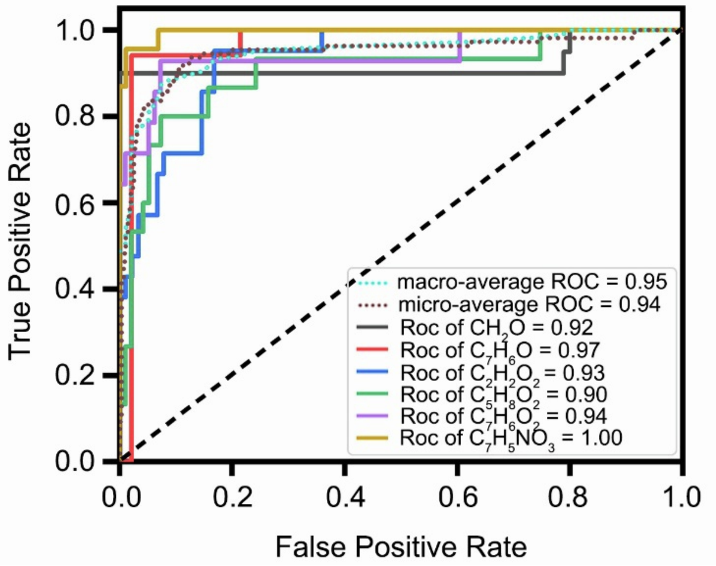

Figure S8. ROC curves of different aldehyde targets (ppt-level). **Related to Figure 5.**

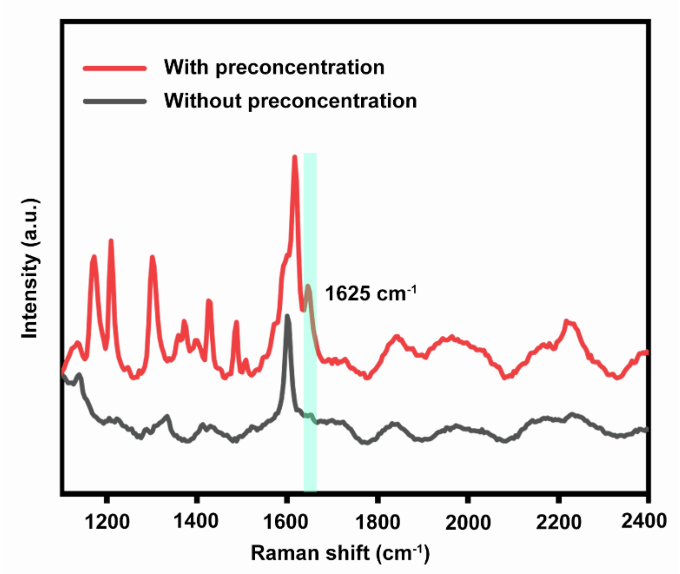

Figure S9. Compare the SERS performances using the same concentration of gas (100 ppt benzaldehyde) with and without preconcentration. **Related to Figure 5.**

Table S1. Formulas and structures of detected aldehydes. **Related to Figure 4.**

Aldehydes name	Formula	Structure
Formaldehyde	CH_2O	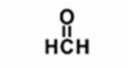
Benzaldehyde	C_7H_6O	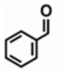
Oxalaldehyde	$C_2H_2O_2$	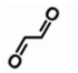
Glutaraldehyde	$C_5H_8O_2$	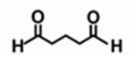
Salicylaldehyde	$C_7H_6O_2$	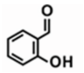
4-Nitrobenzaldehyde	$C_7H_5O_3$	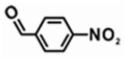

Table S2. Raman assignments of different aldehydes (100 ppb) in SERS-AI detection. **Related to Figure 4.**

4-ATP	CH_2O	C_7H_6O	$C_2H_2O_2$	$C_5H_8O_2$	$C_7H_6O_2$	$C_7H_5O_3$	Assignments
1078	1073	1078	1078	1079	1077	1076	vCS, 7a(a_1)
1141	1138						δCH, 9b(b_2)
1179	1177	1181	1182	1185	1178	1184	δCH, 9a(a_1)
			1223	1213			
				1257			
1322							vCC+δCH, 14b(b_2)
1384	1381						δCH+vCC, 3b(b_2)
1429	1421					1440	vCC+δCH, 19b(b_2)
1593	1585	1588	1578	1578	1568<br>1582	1569<br>1584	vCC, 8b(b_2)
			1632	1627	1610	1652	vCN

v-Stretching vibration, δ-Deformation vibration

Assignments for the SERS spectra of 4-ATP are referenced from other research.[1-3]

Table S3. Raman assignments of different aldehydes (100 ppt) in microfluidic-SERS-AI detection. **Related to Figure 5.**

4-ATP	CH_2O	C_7H_6O	$C_2H_2O_2$	$C_5H_8O_2$	$C_7H_6O_2$	$C_7H_5O_3$	Assignments
1078	1075	1076	1077	1075	1076	1076	*v*CS, 7*a*(*a*1)
1141	1139		1139	1141	1139	1140	*δ*CH, 9*b*(*b*2)
		1166	1157		1163		
1179	1175			1178	1179	1173	*δ*CH, 9*a*(*a*1)
1322							*v*CC+*δ*CH, 14*b*(*b*2)
1384	1387			1390	1389	1387	*δ*CH+*v*CC, 3*b*(*b*2)
1429	1440			1433	1433		*v*CC+*δ*CH, 19*b*(*b*2)
		1552	1522			1558	
1593	1585	1574	1575<br>1586	1588	1587	1588	*v*CC, 8*b*(*b*2)
			1625				*v*CN

v-Stretching vibration, *δ*-Deformation vibration

Assignments for the SERS spectra of 4-ATP are referenced from other research.[1-3]

References

1. Moskovits, M., and Suh, J. S. (1984). Surface selection rules for surface-enhanced Raman spectroscopy: calculations and application to the surface-enhanced Raman spectrum of phthalazine on silver. J. Phys. Chem. *88*, 5526-5530.

2. Zheng, J., Li, X., Gu, R., and Lu, T. (2002). Comparison of the surface properties of the assembled silver nanoparticle electrode and roughened silver electrode. J. Phys. Chem. B. *106*, 1019-1023.

3. Wang, Y., Chen, H., Dong, S., and Wang, E. (2006). Surface enhanced Raman scattering of p-aminothiophenol self-assembled monolayers in sandwich structure fabricated on glass. J. Phys. Chem. *124*, 074709.
